# Protective Effects of Kelulut Honey on Bone Strength and Marrow Adiposity in Rats Fed with High-Carbohydrate High-Fat Diet

**DOI:** 10.7150/ijms.115978

**Published:** 2025-08-16

**Authors:** Sophia Ogechi Ekeuku, Khairun-Nisa Hashim, Jen Kit Tan, Michelle Yee Min Fang, Nur Zuliani Ramli, Khairul Anwar Zarkasi, Kok-Yong Chin, Fairus Ahmad

**Affiliations:** 1 Department of Pharmacology, Faculty of Medicine, Universiti Kebangsaan Malaysia, 56000 Cheras, Malaysia.; 2 Department of Anatomy, Faculty of Medicine, Universiti Kebangsaan Malaysia, 56000 Cheras, Malaysia.; 3 Department of Biochemistry, Faculty of Medicine, Universiti Kebangsaan Malaysia, 56000 Cheras, Malaysia.; 4 Department of Anatomy, Faculty of Medicine, Universiti Teknologi MARA, 47000 Sungai Buloh, Malaysia.; 5 Biochemistry Unit, Preclinical Department, Faculty of Medicine & Defence Health, Universiti Pertahanan Nasional Malaysia, Sungai Besi Camp, 57000 Kuala Lumpur, Malaysia.

**Keywords:** antioxidant, marrow adiposity, osteopenia, osteoporosis, skeleton

## Abstract

**Background/Objectives:** Metabolic syndrome (MetS) may increase the risk of osteoporosis. This study examines the effects of Kelulut honey, previously shown to prevent MetS, on bone health in male rats fed a high-carbohydrate high-fat (HCHF) diet.

**Methods:** Chemical profiling of the honey was performed using liquid chromatography-tandem mass spectrometry methods. For the *in vivo* experiment, male Wistar rats were divided into three groups (n=6/group). The normal control group was fed standard chow, while the HCHF groups were given an HCHF diet for 16 weeks. During the final eight weeks, one HCHF group received Kelulut honey (1 g/kg body weight/day). Bone density, biomechanics, histomorphometry, redox markers, and expression of genes relevant to bone cell differentiation were analysed at the end of the study.

**Results:** Chemical profiling of the honey revealed a unique array of bioactive compounds and oligosaccharides. HCHF diet decreased bone displacement and strain, but increased stiffness compared to controls. Bone marrow adipocyte number also increased with HCHF diet. Kelulut honey improved displacement and strain in HCHF-fed rats and reduced bone marrow adipocyte number. *Pparg* gene expression was significantly lower with HCHF diet. However, no significant differences in bone density, structural and cellular histomorphometric indices, glutathione levels, antioxidant enzyme activities and gene expression of *Rankl*, *Opg*, *Ocn*, *Ctsk* and *Runx2* were observed between groups.

**Conclusion:** HCHF diet compromises bone mechanical strength and bone marrow adiposity. Kelulut honey inverses these negative skeletal changes. These findings suggest Kelulut honey's potential protective role against MetS-related bone health issues, warranting further investigation into its mechanisms.

## Introduction

Metabolic syndrome (MetS) is a cluster of conditions, including abdominal obesity, abnormal lipid levels, elevated blood sugar, and hypertension, which significantly increase the risk of cardiovascular disease and type 2 diabetes 1. This condition affects individuals of all ages, genders, and ethnicities, with worldwide prevalence rates ranging from 12.5% [95 % confidence interval (CI): 10.2-15.0] to 31.4% (29.8-33.0), posing a substantial public health challenge 2. Notably, the components of MetS have been linked to factors associated with osteoporosis development 3. Bone loss has been detected in rats with MetS induced by a high-carbohydrate high-fat (HCHF) diet in previous studies 4, 5.

Bone remodelling is a delicate physiological process that maintains skeletal integrity by balancing bone resorption and formation. The differentiation of osteoclasts, which mediate bone resorption, is stimulated through receptor activator of nuclear factor kappa-B (RANKL) and inhibited by osteoprotegerin (OPG). Osteoblasts secrete both of these molecules 6. During bone resorption, osteoclasts express cathepsin K (CTSK), an enzyme responsible for degrading the bone matrix 7. On the other hand, osteoblasts drive bone formation by synthesising bone matrix proteins, including osteocalcin (OCN), a marker of mature osteoblasts 8. The transcription factor, runt-related factor 2 (RUNX2), is essential for osteoblast differentiation, guiding progenitor cells toward the osteoblastic lineage 9. Meanwhile, peroxisome proliferator-activated receptor gamma (PPARG) expression drives mesenchymal stem cells to differentiate into adipocytes 10. The balance between these molecular markers ensures proper bone remodelling, and disruptions in their expression can contribute to skeletal disorders such as osteoporosis.

Altered redox status and bone marrow adiposity are among the postulated mechanisms of bone loss in MetS 11, 12. Patients with MetS often experience systemic oxidative stress due to mitochondrial dysfunction, impaired antioxidant defence and damage to macromolecules 13. Oxidative stress stimulates osteoclast formation and bone resorption, while suppressing osteoblast formation and bone building 14. Bone marrow adiposity is observed in patients with MetS. Since osteoblasts and adipocytes originate from the same pool of mesenchymal stem cells, the increased adipocyte formation in bone marrow might deplete the stem cell pool for osteoblast formation, thereby reducing the bone-forming capacity of the tissue 15.

Kelulut honey (KH), a functional food produced by stingless bees (*Trigona* species) 16, is rich in polyphenols, including flavonoids and phenolic acids, compared to other commercially available honey 17, 18. These bioactive compounds exhibit antioxidant properties through diverse mechanisms, such as free radical scavenging and metal chelation 19. The synergistic interactions between honey's inherent antioxidant capacity and the body's endogenous antioxidant enzymes contribute to the neutralisation of reactive oxygen species and the induction of an antioxidant response 20, 21.

Preclinical studies have demonstrated honey's potential to inhibit osteoclast formation, a key process in bone degradation. This protective effect is attributed to the presence of flavonoids, such as kaempferol and quercetin, which have been shown to suppress osteoclastic differentiation and bone resorption by downregulating nuclear factor kappa B (NF-κB) and inducing osteoclast apoptosis 22, 23. Furthermore, flavonoids have been shown to prevent the formation of multinucleated osteoclasts and reduce the expression of osteoclastic differentiation markers 24. Besides, previous research has indicated that honey supplementation can improve metabolic parameters by decreasing body fat percentage, triglyceride levels, blood pressure, and adipocyte hypertrophy in a rat model of high-carbohydrate, high-fat diet-induced obesity 25.

Ekeuku *et al.* 26 previously showed that Kelulut honey supplementation prevented the increase in osteoclasts but not bone microstructural and biomechanical deterioration in male rats with HCHF-induced MetS. Data on bone densitometric changes and mechanisms of action induced by Kelulut honey were lacking. Thus, this study evaluated the impacts of Kelulut honey on MetS-induced osteoporosis in male rats fed with HCHF on bone densitometry, biomechanical strength, microstructural and cellular histomorphometry. In particular, the effects of Kelulut honey on skeletal redox status, bone marrow adiposity and expression of genes related to bone remodelling were examined to explain the mechanism of action of Kelulut honey.

## Materials and Methods

### Chemical profiling of honey

For the extraction of metabolites, 100 mg of honey was mixed with 1 mL of a methanol:water solution in a 1:1 ratio (methanol, cat no: A456-4; Fischer Scientific). The mixture underwent vortexing for 30 seconds, followed by sonication for 30 minutes using a Texsonic bench-top ultrasonic bath (TUC-P40H, Taipei City, Taiwan). The sonicator was operated at full power, with a frequency of 37 kHz and a pulse setting of 120 arbitrary units (AU), while the temperature was maintained at 4°C using an ice pack. After sonication, the sample was filtered through a 0.2 µm PTFE syringe membrane (15 mm diameter; #AF0-2202-52; Phenomenex, Torrance, USA) into a vial (AR0-9974-13-C; Phenomenex). Vortexing was performed with a VM3 vortexer (Ingenieurbüro CAT, M. Zipperer GmbH, Ballrechten-Dottingen, Germany) at a maximum speed of 2,800 revolutions per minute (rpm) with an orbital motion.

The global metabolomics profiling of the samples was carried out using ultra-high-performance liquid chromatography-tandem mass spectrometry (UHPLC-MS/MS), following a previously established protocol with minor modifications 27. The liquid chromatography was performed on a Dionex UltiMate 3000 system (Thermo Scientific Waltham, USA) connected to an Orbitrap MS/MS (Q Exactive HF; Thermo Scientific) via a heated electrospray ionisation (HESI) probe. Calibration of the instrument was achieved using Pierce LTQ ESI Positive (#88323; Thermo Scientific) and Negative Ion (#88324; Thermo Scientific) Calibration Solutions. The separation of compounds was accomplished using a C18 column (1.7 μm particle size, 100 mm length, 2.1 mm diameter; Synchronis, #97102-102130; Thermo Scientific). The conditions for LC were set as follows: the column temperature was maintained at 55 °C, the autosampler temperature at 10 °C, the injection volume at 2 μL, and the flow rate at 0.45 mL/min. Solvent A was water (W6-4; Fisher Scientific, Hampton, USA), while solvent B was acetonitrile (ACN, A955-4; Fisher Scientific), both containing 0.1% formic acid (A117-50; Fisher Scientific) (v/v). A 22-minute elution gradient was implemented: at 0 and 1 minutes, 0.5% B; at 16 and 20 minutes, 99.5% B; and at 22 minutes, returning to 0.5% B.

The HESI was operated separately in both positive and negative ionisation modes. The tuning of the HESI MS source involved adjusting the sweep gas flow rate to 50 AU, the auxiliary gas flow rate to 18 AU, and the capillary temperature to 320 °C. The S-lens level was set at 55 AU, the auxiliary gas heater temperature at 300 °C, and the spray voltage was configured to 3.5 kV for positive mode and 3.0 kV for negative mode. Mass spectra were collected using a full MS scan followed by a data-dependent tandem mass spectrometry (ddMS2) scanning method, as specified in Xcalibur 4.2.27 software (Thermo Scientific). The full MS scan was recorded at a resolution of 60,000 across a mass/charge (m/z) range of 100-1,000, while the ddMS2 scan was captured at a resolution of 15,000 with stepped normalised collision energies of 20, 40, and 60 AU.

The raw data generated from the LCMS analysis underwent pre-processing using MS-DIAL (v5.2.240424.3) 28. The tolerances for MS1 and MS2 were set at 0.01 and 0.025 Da, respectively, with a minimum peak height threshold established at 30,000 amplitude and a mass slice width of 0.05 Da. The identification of metabolites was conducted automatically by the software, utilising the MS2 public database from authentic standards available on the MS-DIAL website (version 17; systemsomicslab.github.io). Features identified in blank samples were excluded based on a sample maximum/sample average signal ratio of less than 5-fold change. Additionally, annotated features were manually reviewed by the researcher (J.K.T) to assess MS1 peak shape and ensure proper matching with MS2 reference peaks.

### Preparation of KH for animal treatment

Raw honey (KH) sourced from the stingless bee species *Heterotrigona itama* was acquired from a local apiary located in Gombak, Selangor, Malaysia. It was kept in a glass container at a temperature of 4°C. Prior to administration by oral gavage, KH was mixed with distilled water at an equal ratio of 1:1.

### HCHF preparation

The HCHF diet was formulated with the following components per kilogram: 395 g of sweetened condensed milk (Fraser & Neave Holdings Bhd., Kuala Lumpur, Malaysia), 200 g of ghee (Enrico's Pure Ghee, Raviraj Sdn. Bhd., Penang, Malaysia), 175 g of D-(-)-fructose (Emprove® Essential, Merck, Darmstadt, USA), 155 g of powdered rat chow (Gold Coin Feedmills (M) Sdn. Bhd., Selangor, Malaysia), 25 g of a salt mixture from Hubble, Mendel, and Wakeman (MP Biomedicals, California, USA), and 50 mL of water. Additionally, the drinking water for the HCHF groups was supplemented with a solution containing 25% fructose (Merck). This dietary composition has been shown to effectively induce MetS in rats after a duration of 16 weeks. 25. All rats had unrestricted access to feed and water.

### Animals

Twenty-four adult male Wistar rats (mean weight 275 g) were procured from the Laboratory Animal Resource Unit, Universiti Kebangsaan Malaysia. Following a two-week acclimatisation period, the animals were individually housed in a controlled environment at the Anatomy Department, Faculty of Medicine, Universiti Kebangsaan Malaysia. This environment was maintained at a constant temperature of 25 ± 3°C and a 12-hour light-dark cycle. All experimental procedures adhered to the ethical guidelines for animal research established by Universiti Kebangsaan Malaysia and were approved by the Universiti Kebangsaan Malaysia Animal Ethics Committee (approval code: ANAT/FP/2020/FAIRUS AHMAD/23-SEPT./1126-OCT.-2020-SEPT-202).

### Sample size calculation

Based on the effects of HCHF diet on displacement 26, the sample size calculation was performed using G*Power 3.1.9.7 (University of Düsseldorf, Düsseldorf, Germany) using the input: effect size=1.14, α error=0.05, power=0.9, number of groups =3. A total sample size of 15 or 5/group was obtained. It was raised to 18 or 6/group in case of unexpected sickness/death of the animals during the experiment.

### Study design

A total of 18 adult male Wistar rats were randomly assigned to three groups (n=6/group): a normal control group, a negative control group (HCHF), and a Kelulut honey intervention group (HCHF+K). Randomisation was done using random numbers generated by the Statistical Package for Social Sciences (SPSS) version 26 (IBM, Armonk, NY, USA). The normal control group was provided standard rodent pellets (GoldCoin, Klang, Malaysia) and tap water *ad libitum*. The negative control and honey groups were given the HCHF diet and 25% fructose drinking water for 16 weeks. During the final eight weeks of the study, the honey group received Kelulut honey via oral gavage (1 g/kg body weight/day), while the other two groups received distilled water. The establishment of MetS in this study has been described in a previous publication 25. At the end of the experimental period, animals were euthanised using a ketamine/xylazine/Zoletil mixture (0.3 mL/100 g body weight). Both femurs and tibias were subsequently excised and cleaned of soft tissue. Left femurs and tibias were stored at -80°C for biochemical analysis, while right femurs and tibias were preserved in 10% neutral buffered formalin for histological examination.

### Blinding

Different researchers performed animal treatment and bone sample assessment. Researchers responsible for bone sample assessment were blinded to the grouping of the rats until the statistical analysis stage.

### Bone density assessment

Left femurs harvested were oriented vertically for analysis. Femoral bone area, mineral density (BMD) and content (BMC) were assessed via dual-energy X-ray absorptiometry utilising a Discovery Wi Bone Densitometer (Hologic, MA, USA) in high-resolution mode.

### Bone histomorphometry

The right femurs, free of soft tissue, were longitudinally bisected. One half was decalcified in 10% ethylenediaminetetraacetic acid (EDTA) for 30 days at room temperature. Following decalcification, this half was embedded in paraffin, sectioned at 5 μm using a microtome (Leica RM2235, Nussloch, Germany), and stained with haematoxylin and eosin (H&E) following deparaffinisation procedures (xylene washes and graded alcohol dehydration/rehydration). Sections were then dehydrated and cleared (graded alcohols and xylene) and examined under a light microscope (Zeiss Primo Star, Germany) at 100 × magnification using Zen 2.6 lite software for image capture. The undecalcified femoral half was embedded in polymethyl methacrylate and sectioned at 8 μm thickness using a microtome. Sections were stained using the von Kossa method, involving acetone washes, graded alcohol rehydration, and incubation with 1% silver nitrate under UV light for 20 minutes, followed by rinsing and incubation with 2.5% sodium thiosulfate for 5 minutes. Stained sections underwent dehydration (graded alcohols) and clearing (diethyl ether) before examination under a light microscope (Zeiss Primo Star, Germany) at 20 × magnification and image capture using Zen 2.6 lite software. Bone morphometry included assessment of bone volume/total volume (BV/TV), trabecular thickness (Tb.Th), number (Tb.N), and separation (Tb.Sp).

The left femur was similarly dissected and bisected. One half underwent decalcification in 10% EDTA for 72 hours at 37°C. Following rinsing and overnight storage at -80°C, tissues were embedded in OCT compound. A cryostat microtome (HM525 NX Cryostat, Thermo Scientific, Waltham, USA) was used to section the frozen tissues at 5 μm thickness onto Polysine® glass slides. Sections were stained with Oil Red O for 10 minutes and counterstained with haematoxylin. Images were captured using a light microscope (BX53, Olympus, Tokyo, Japan). Adipocyte number per tissue area (N.Ac/T.Ar) was quantified using ImageJ 1.52a software (National Institutes of Health, Bethesda, USA) by analysing the red colour-stained area.

### Biomechanical assessment

To assess the mechanical properties of the left tibia, a three-point bending test was performed at the mid-diaphyseal region. Following thawing to room temperature, tibiae were weighed, and their dimensions were recorded. Each bone was positioned with its anterior surface downward on two 10-mm supports and subjected to a centrally applied load at a crosshead speed of 5 mm/min until fracture. A Shimadzu Universal Testing Machine (Autograph AGS-X 500N) was utilised for load application. Load, displacement, stress, and strain values were determined using Trapezium X software. Stiffness was calculated as the ratio of load to displacement, while Young's modulus was derived from the stress-strain relationship.

### Skeletal redox status

Tissue samples from the left tibia were homogenised in either Tris or phosphate buffer according to the methodology outlined by Ekeuku *et al.* 29. The resulting homogenates were subjected to centrifugation under specified conditions, and the supernatant was subsequently stored at -80°C.

Total protein content was determined using the Bradford assay with bovine serum albumin as a standard. Protein concentrations in the tissue homogenates were calculated from absorbance measurements at 595 nm.

Levels of glutathione (GSH) and malondialdehyde (MDA), and activities of superoxide dismutase (SOD) and catalase (CAT), were assessed using modified protocols adapted from Ekeuku *et al.* 29 and Khare *et al.* 30. GSH concentrations in Tris homogenates were quantified using a spectrophotometric assay. Briefly, 60 μL of homogenate was added to a 96-well plate containing Ellman's reagent and phosphate buffer. After incubation at 25°C for 5 minutes, and absorbance measurement at 412 nm, GSH concentrations were calculated and expressed as mmol/mg protein.

A 96-well plate was used to prepare a reaction mixture for SOD, which contained sodium carbonate, nitroblue tetrazolium, EDTA, and Tris tissue homogenate. Hydroxylamine hydrochloride was then added, and after a two-minute incubation, the absorbance at 560 nm was measured to determine SOD activity, which was expressed as units per mg of protein.

CAT activity was evaluated in a 96-well plate by mixing 190 μL of 0.05 M phosphate buffer (pH 8), 10 μL of phosphate-buffered bone tissue homogenate, and 100 μL of 0.03 M hydrogen peroxide. The absorbance of the reaction mixture was recorded at 612 nm every 30 seconds for a duration of 2 minutes. Catalase activity was expressed as the rate of substrate decomposition (mmol/mg protein).

MDA levels were determined using a thiobarbituric acid reactive substances assay. Briefly, tissue homogenates were treated with trichloroacetic acid and centrifuged. The supernatant was then reacted with thiobarbituric acid and incubated at 95°C. The absorbance of the resulting pink chromophore was measured at 532 nm, and MDA levels were calculated and expressed as nmol/g wet tissue.

### Gene expression analysis

The expression levels of *Opg*, *Ocn*, *Rankl*, *Pparg*, *Runx2*, and *Ctsk* mRNA were analysed using real-time reverse transcription polymerase chain reaction (RT-PCR). Femoral tissue samples were preserved in RNAwait (Solarbio, Beijing, China) at a 1:10 ratio. Total RNA was extracted following the manufacturer's protocol using a spin column-based method (E.Z.N.A Total RNA Kit I, Omega Biotek, Norcross, USA), and its concentration was assessed by measuring absorbance at 260 nm with a Nanodrop spectrophotometer (Thermo Fisher, Waltham, USA). Complementary DNA (cDNA) was then synthesised from the isolated RNA using the OneScript Plus cDNA Synthesis Kit (ABMgood, Richmond, Canada). The reaction was carried out in a thermocycler at 55 °C for 15 minutes, followed by enzyme inactivation at 85 °C for 5 seconds. RT-PCR amplification was performed using BlasTaq™ 2X qPCR MasterMix (ABMgood, Richmond, Canada), incorporating the cDNA template, gene-specific primers (Integrated DNA Technologies, Coralville, USA) (Table 1), and nuclease-free water (Vivantis Technologies, Shah Alam, Malaysia) in a final volume of 20 µL. The reaction was run on the Mic PCR System (Bio Molecular System, Upper Coomera, Australia) under the following conditions: an initial denaturation at 95 °C for 3 minutes, followed by 45 cycles of 95 °C for 3 seconds and 60°C for 30 seconds.

The relative gene expression levels were calculated using the 2^-ΔΔCT^ method. Cycle threshold (CT) values were derived from real-time PCR analysis. Initially, the CT values for the target gene were normalised against the reference gene, *Gadph*, employing the formula ΔCT = CT of the target gene - CT of Gadph. Following this, the ΔCT values for the treatment groups were further normalised to those of the sham control group using the formula ΔΔCT = ΔCT treatment group - mean ΔCT sham control.

### Statistical analysis

Statistical analysis was conducted using version 26 of the Statistical Package for Social Sciences (SPSS) (IBM, Armonk, USA). All animal data points were included in the analysis. The Shapiro-Wilk test was employed to assess data normality, confirming that all datasets followed a normal distribution. A one-way analysis of variance with Tukey's pairwise comparison was utilised to evaluate mean differences among the study groups, with statistical significance set at p < 0.05.

## Results

### Chemical characteristics of the honey

**Figure 1:**
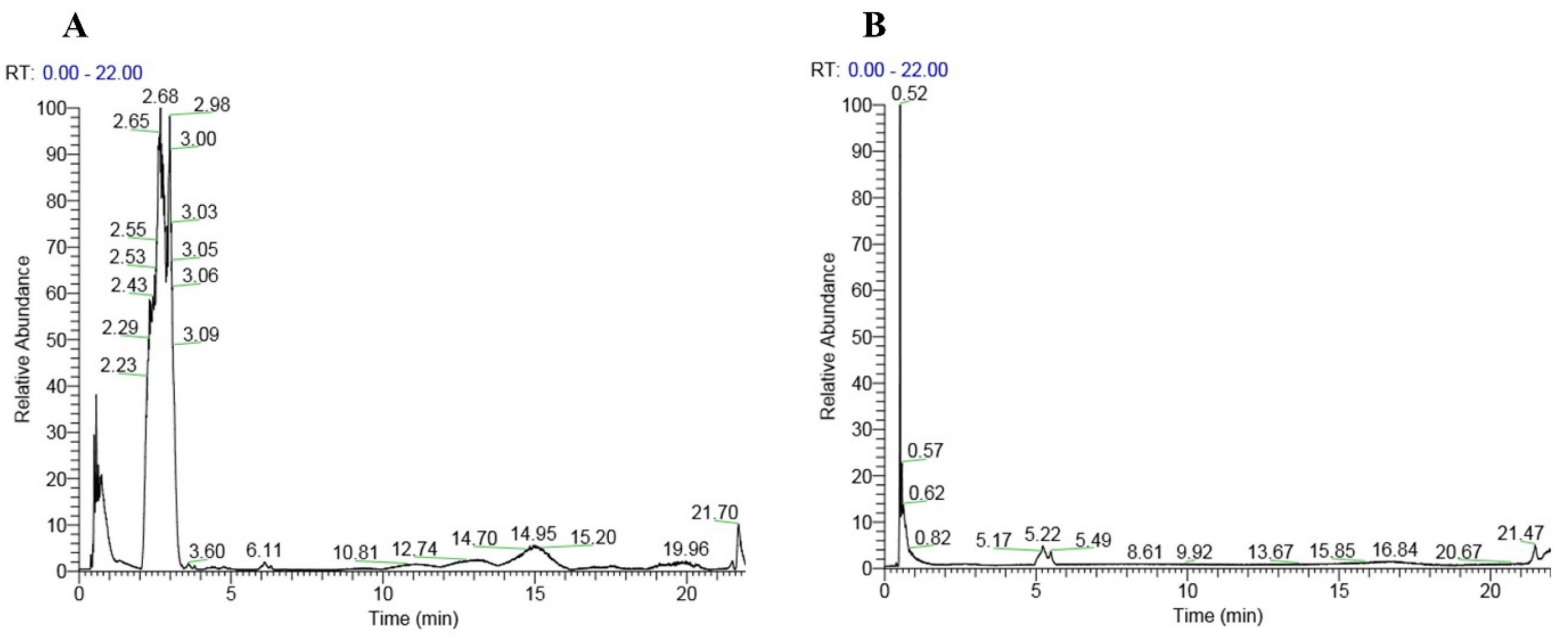
Chromatogram of the honey showing peaks in positive mode (A) and negative mode (B).

The analysis of the honey sample using liquid chromatography-tandem mass spectrometry (LC-MS/MS) revealed the presence of a diverse array of compounds, ranging from small molecules such as gamma-aminobutyric acid with a molecular weight of 103.063 Da to larger compounds like stachyose with a molecular weight of 666.221 Da. Notably, several amino acids were detected, including histidine and phenylalanine. Additionally, complex oligosaccharides unique to honey, such as melezitose, raffinose, stachyose and isomaltulose, were also present. Secondary metabolites, including indole-3-carboxaldehyde and kojic acid, were also detected. The chromatogram of the peaks in positive and negative modes is shown in Figure 1. Table 2 lists the compounds identified in the honey sample.

### Bone density assessment

**Figure 2:**
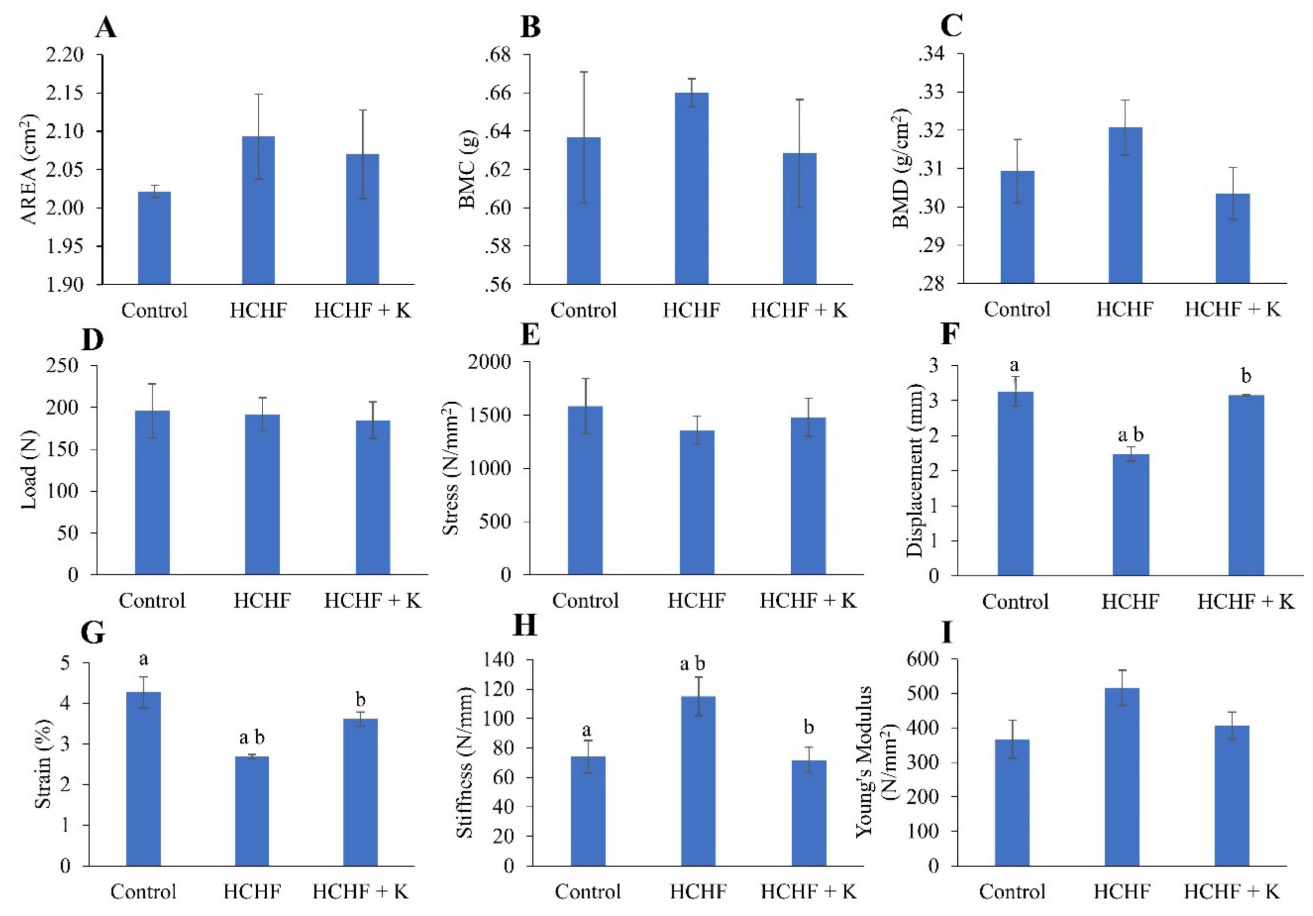
Bone densitometric data obtained by dual-energy X-ray absorptiometry (A-C) and biomechanical strength assessment (D-I) results of the rats (n=6/group) during the experiment. The data are expressed as mean ± standard error. One-way ANOVA, followed by Tukey's post hoc test, was employed to assess the differences among the groups. Groups sharing the same letters are significantly different from each other (p<0.05). Abbreviations: HCHF, high-carbohydrate high-fat; HCHF+K, Kelulut honey; BMD, bone mineral density; BMC, bone mineral content.

Administration of the HCHF diet alone or subsequent treatment with Kelulut honey did not alter the bone area, BMC and BMD of the left femur (p > 0.05) (Fig. 2A-C). Similarly, load, stress and Young's Modulus were not altered with HCHF and Kelulut honey supplementation (p > 0.05) (Fig. 2D, E, I). However, the HCHF diet caused a reduction in displacement and stress, as well as an increase in stiffness (p < 0.05 vs the normal control). These changes were reversed by Kelulut honey supplementation (p < 0.05 vs HCHF) (Fig. 2 F, G).

### Bone histomorphometry

**Figure 3:**
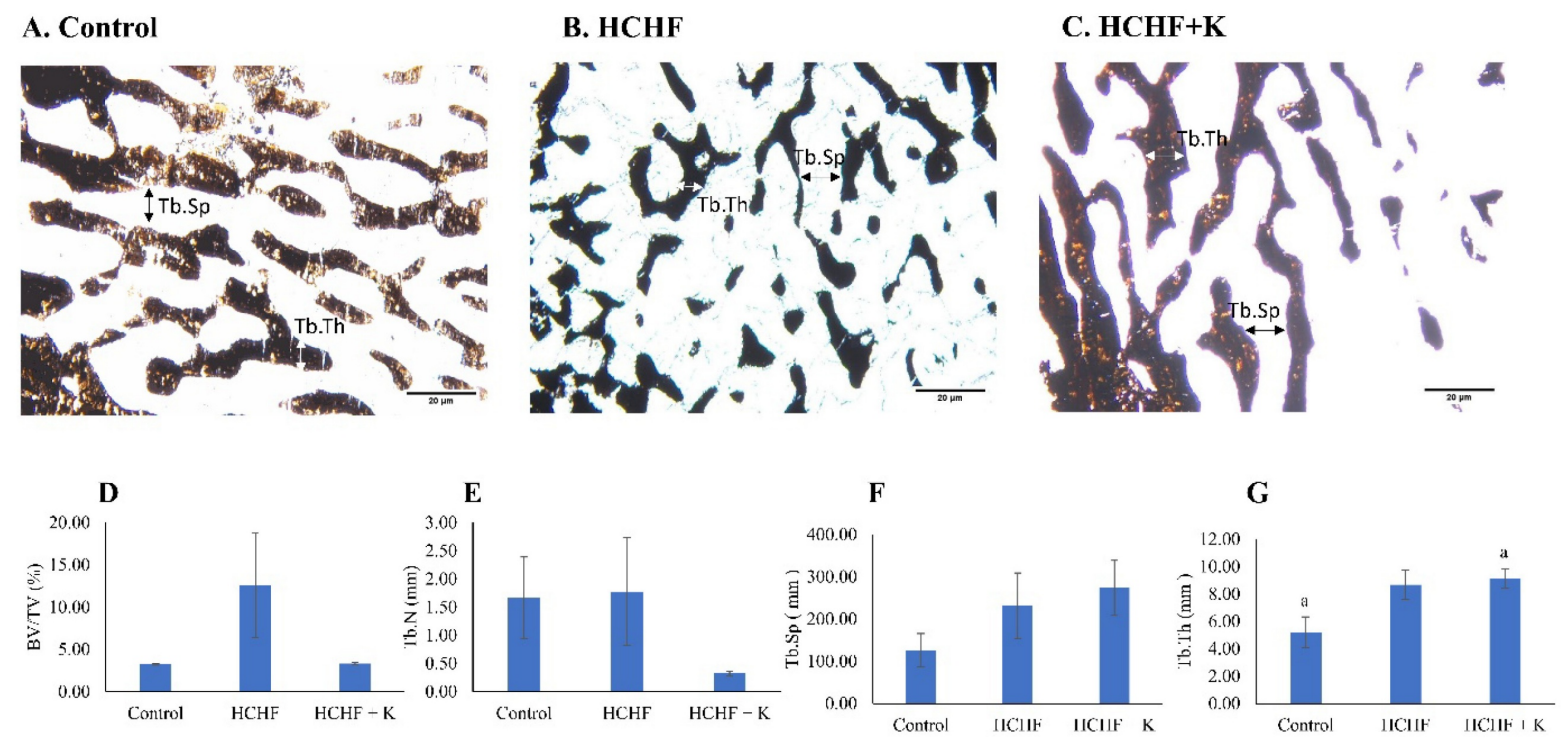
Micrograph of femur sections (100 × magnification) stained using the von Kossa method/silver nitrate (A-C) for each group (n=6/group). The white arrow indicates Tb.Th, while the black arrow indicates Tb.Sp. The structural histomorphometric indices of the femur evaluated are BV/TV (D), Tb.N (E), Tb.Sp (F), and Tb.Th (G). The quantitative data are expressed as mean ± standard error. One-way ANOVA, followed by Tukey's post hoc test, was employed to assess the differences among the groups. Groups sharing the same letters are significantly different from each other (p<0.05). Abbreviations: HCHF, high-carbohydrate high-fat; HCHF+K, Kelulut honey; BV/TV, bone volume/total volume; Tb.Th, trabecular bone thickness; Tb.N, trabecular bone number; Tb.Sp, trabecular bone separation.

Silver nitrate stained the trabecular bones dark brown (Fig. 3A-C). Histomorphometry evaluation of bone microstructure revealed no significant differences in BV/TV, Tb.N and Tb.Sp among the study groups (p > 0.05) (Fig. 3 D-G). However, Tb.Th values were significantly higher in the Kelulut honey-supplemented group than in the control group (p < 0.05).

**Figure 4:**
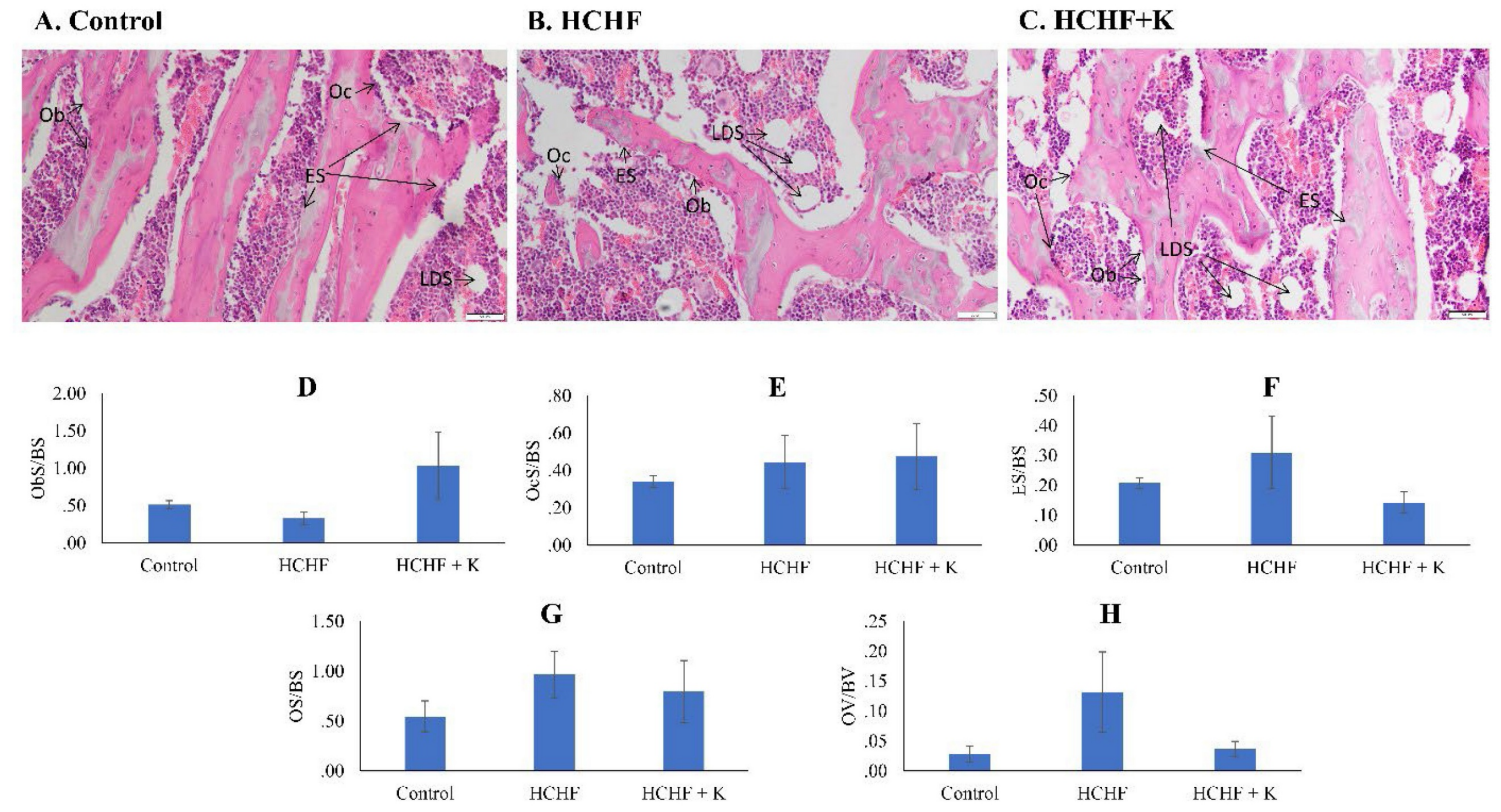
Micrograph of H & E- (A-C) (200 × magnification) femur sections for each study group (n=6/group). Cellular histomorphometric indices of femur bone evaluated are Ob.S/BS (D), Oc.S/BS (E), ES/BS (F), OS/BS (G) and OV/BV (H). The data are expressed as mean ± standard error. One-way ANOVA, followed by Tukey's post hoc test, was employed to assess the differences among the groups. Abbreviations: Ob.S/BS, osteoblast surface; Oc.S/BS, osteoclast surface; ES/BS, eroded surface; OS/BS, osteoid surface; OV/BS, osteoid volume; Ob, osteoblast; ES, eroded surface; LDS, lipid droplet shape; HCHF, high-carbohydrate high-fat; HCHF+K, Kelulut honey.

The H&E slides revealed the bone cells and osteoid on the trabecular surfaces. The HCHF diet and Kelulut supplementation did not induce significant differences in any bone cellular histomorphometry indices at the femur (p > 0.05) (Fig. 4).

**Figure 5:**
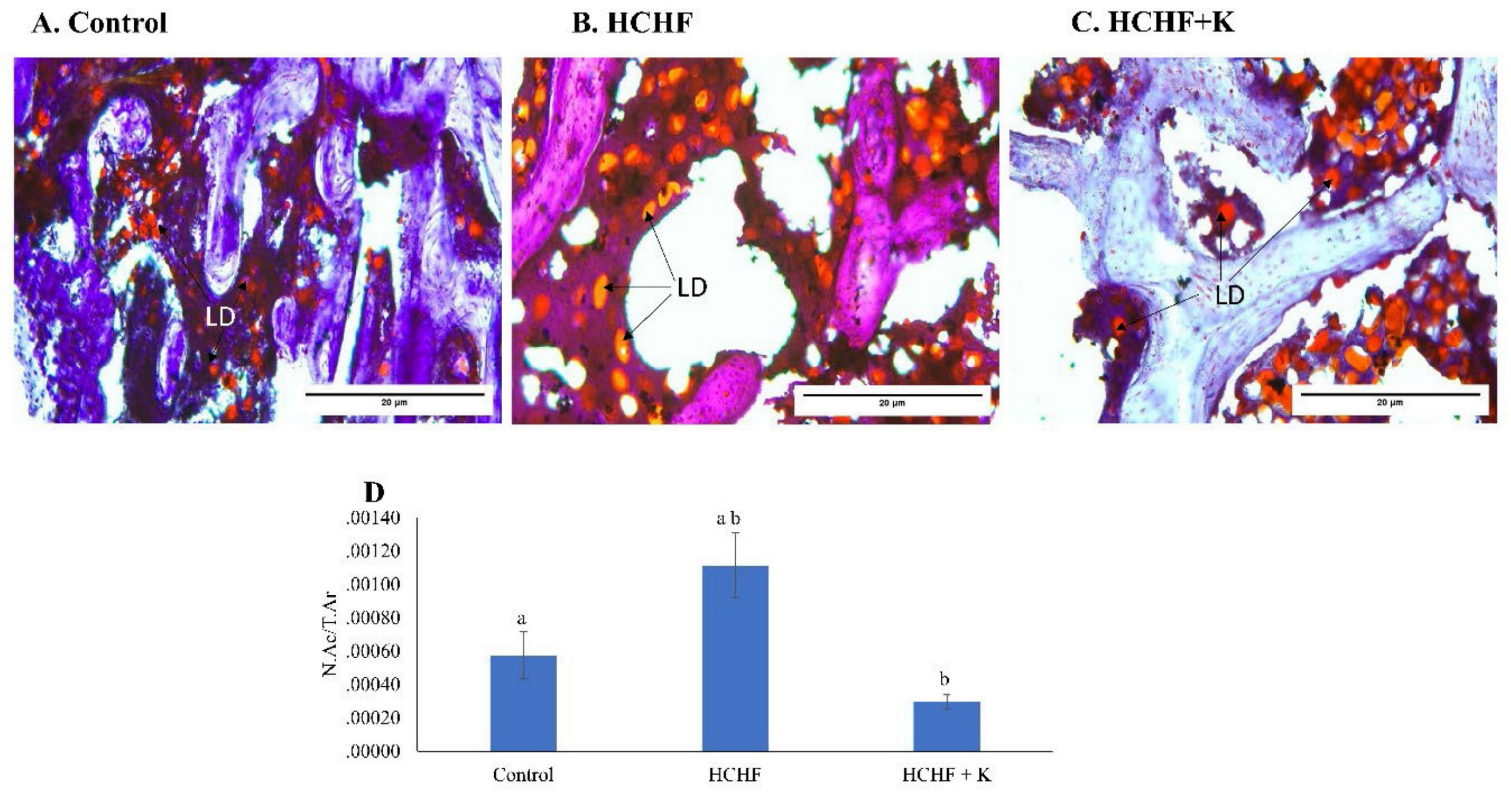
Micrograph of oil red O-stained (A-C) (400 × magnification) femur sections for each study group (n=6/group). The back arrows show lipid droplets. The data are expressed as mean ± standard error. One-way ANOVA, followed by Tukey's post hoc test, was employed to assess the differences among the groups. Groups sharing the same letters are significantly different from each other (p<0.05). Abbreviations: N.Ac/T.Ar, number of adipose cells/total bone area; LD, lipid droplets; HCHF, high-carbohydrate high-fat; HCHF+K, Kelulut honey.

Bone marrow examination of H&E micrographs suggested the abundance of lipid droplets in the HCHF-fed rats (Fig. 4A-C). These findings were verified by Oil Red O staining, which specifically labelled the droplets red (Fig. 5A-C). Notably, the HCHF group exhibited a significant increase in the N.Ac/T.Ar ratio (p < 0.001 vs the negative control). Kelulut honey-treated groups reversed this increase (p < 0.001 vs HCHF) (Fig. 5D).

### Skeletal markers of redox status

**Figure 6:**
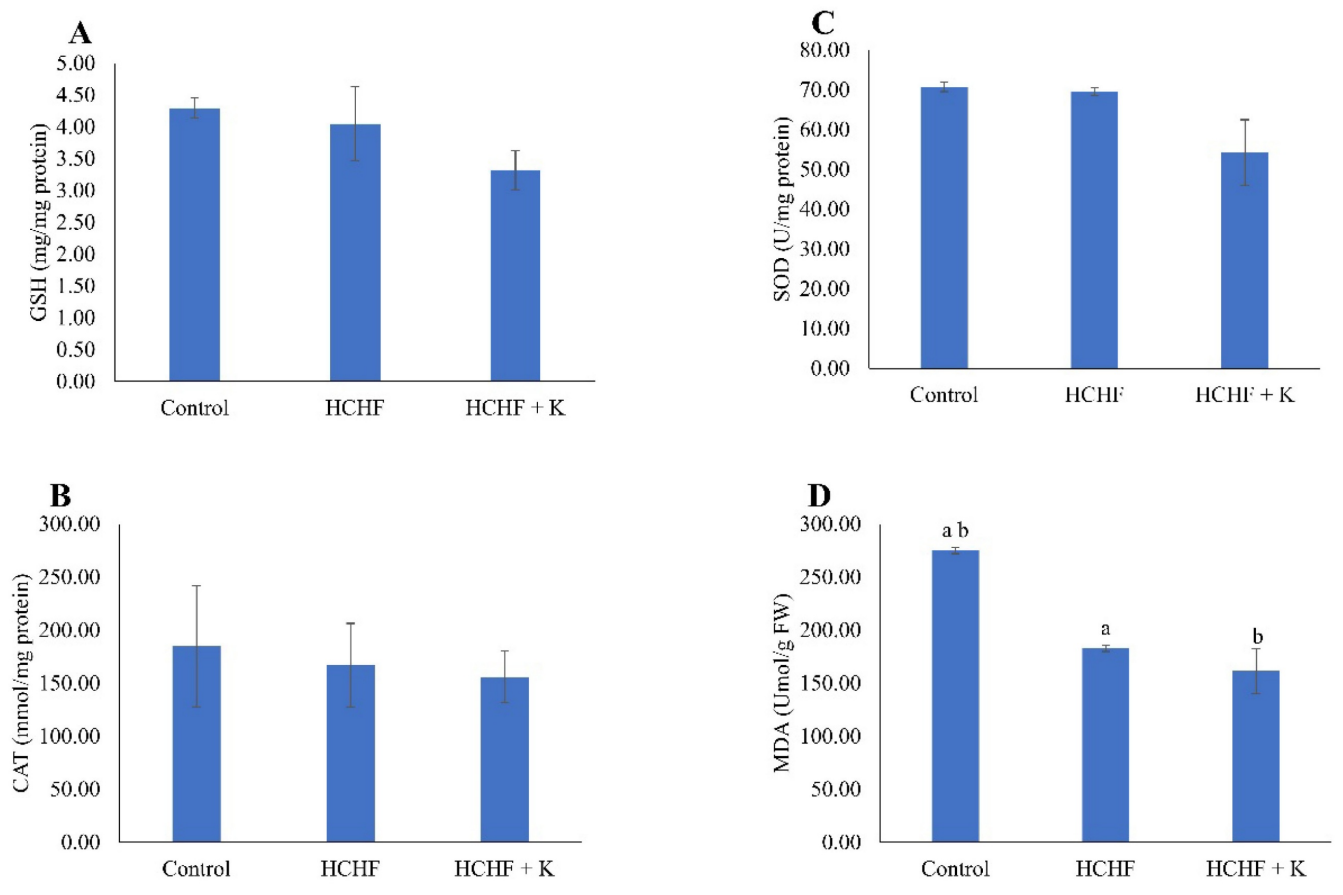
The redox status of the rats is reflected by GSH level (A), CAT activity (B), SOD activity (C) and MDA level (D). The data are reported as mean ± standard error (n=6/group). One-way ANOVA, followed by Tukey's post hoc test, was employed to assess the differences among the groups. Groups sharing the same letters are significantly different from each other (p<0.05). Abbreviations: FW, femur's weight; GSH, glutathione; CAT, catalase; SOD, superoxide dismutase; MDA, malondialdehyde; HCHF, high-carbohydrate high-fat; HCHF+K, Kelulut honey

HCHF diet and Kelulut honey supplementation did not alter the level of GSH, as well as the activities of SOD and CAT (p > 0.05) (Fig. 6A-C). However, MDA levels were lower in the HCHF and honey-supplemented groups than in the control group (p < 0.05) (Fig. 6D).

### Gene expression

**Figure 7:**
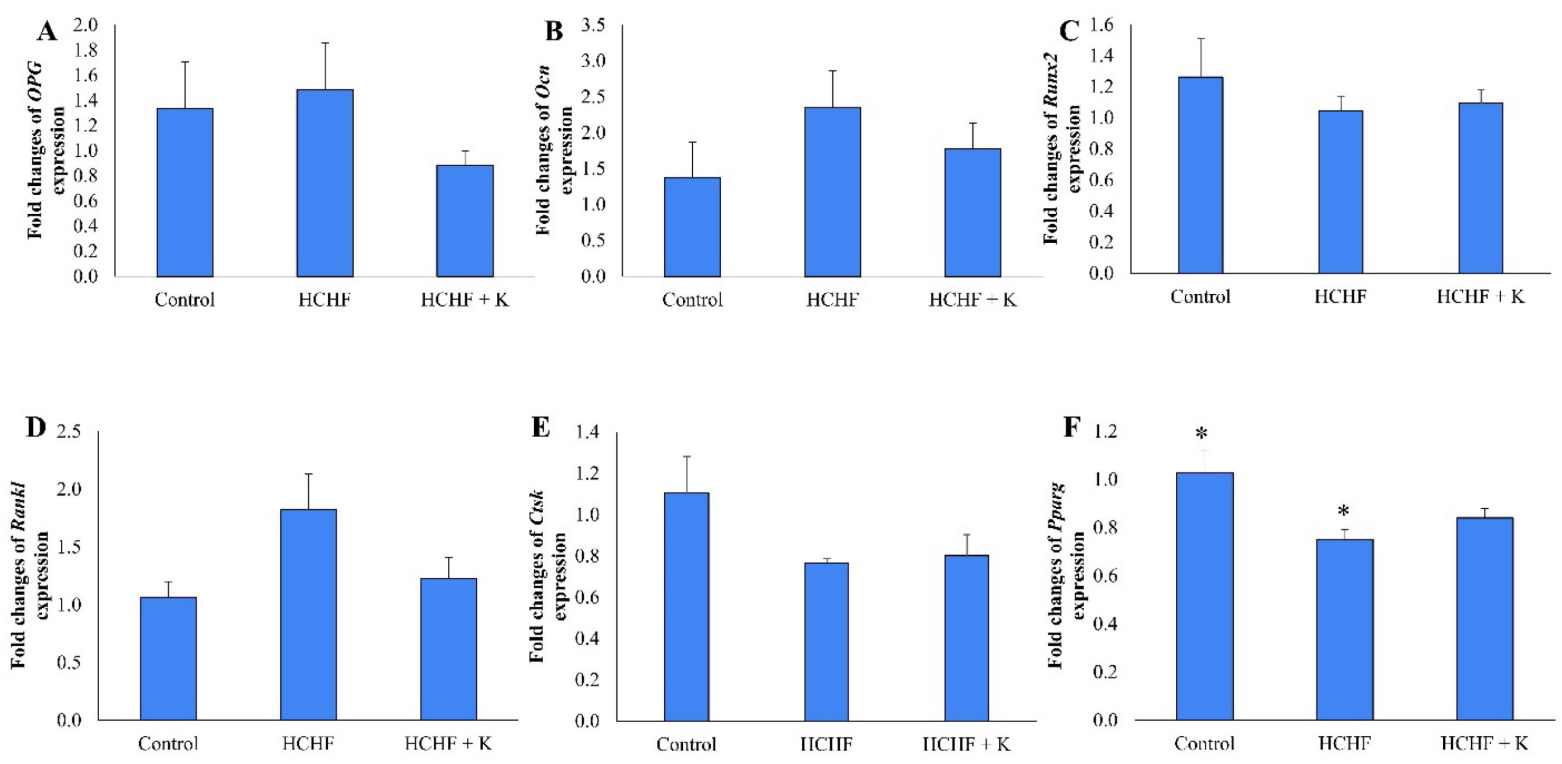
The effect of Kelulut honey on osteogenic-related bone expression. RT-qPCR of Opg (A), Ocn (B), Runx2 (C), Rankl (D), Ctsk (E) and Pparg (F) in HCHF-induced MetS in rats. The data are expressed as mean ± standard error. One-way ANOVA, followed by Tukey's post hoc test, was employed to assess the differences among the groups. * indicates a significant difference (p<0.05) between the marked groups. Abbreviations: HCHF, high-carbohydrate high-fat; HCHF+K, Kelulut honey.

HCHF diet and Kelulut honey supplementation did not alter the expression of *Opg*, *Ocn*, *Runx*, *Rankl* and *Ctsk* (p > 0.05) (Fig. 7A-E). However, *Pparg* expression was downregulated in the HCHF group compared to the control group (p < 0.05). Kelulut honey supplementation did not reverse the effect of HCHF diet on *Pparg* expression (Fig. 7F).

## Discussion

HCHF resulted in the deterioration of bone health in rats, marked by lower displacement and strain. Furthermore, it also increased bone stiffness and bone marrow adiposity. Surprisingly, *Pparg* expression was downregulated by HCHF diet. Kelulut honey reversed these adverse changes caused by the HCHF diet by reducing lipid peroxidation and preventing bone marrow adiposity. Bone microstructural and cellular indices, and gene expression related to osteoblasts and osteoclasts, were not affected by HCHF and Kelulut honey supplementation.

The identification of specific compounds within the honey sample suggests potential beneficial effects on the skeletal system. For instance, histidine has been implicated in hydroxyapatite mineralisation *in vitro*, and its circulating levels, along with alanine, arginine, lysine and glutamine, have been associated with fracture risk reduction 31, 32. The presence of secondary metabolites like indole-3-carboxaldehyde and kojic acid further underscores honey's potential bioactivity. Indole-3-carboxaldehyde has been noted for its anti-inflammatory properties by suppressing the nuclear factor kappa B (NFκB) signalling pathway 33, which could mitigate bone resorption processes. Kojic acid possesses both antioxidant and anti-inflammatory activities, which may protect bone cells from oxidative stress 34. Furthermore, the unique oligosaccharides identified in the honey have been shown to modulate gut microbiota and reduce gut inflammation 35, 36. They can potentially benefit the gut-bone axis. Collectively, these compounds highlight honey's multifaceted potential in promoting bone health.

High-resolution DXA was used to estimate the BMC and BMD of the rats' left femurs. BMC reflects the total mineral content of the specimen, while BMD is calculated by dividing BMC by bone area 37. Although considered a gold standard in diagnosing osteoporosis clinically, DXA may be less sensitive in detecting small changes of BMD and BMC in small animals 38. This reason might explain the lack of significant differences in bone area, BMC, and BMD among the study groups. Our results align with those of Wong *et al.* 4, who observed no differences in BMC or BMD in rats fed an HCHF diet for 20 weeks compared to normal control rats. Similarly, Doucette *et al.* 39 and Fehrendt *et al.* 40 found no significant differences in BMD between rats fed with a high-fat diet and standard rat chow. Thus far, data on the effects of Kelulut honey on bone densitometry have been absent in the literature.

A three-point bending test was performed to assess the biomechanical strength of the bone. Rats fed with the HCHF diet exhibited decreased bone flexibility, as evidenced by reduced displacement and strain, as well as increased stiffness, compared to the control group. These findings are consistent with previous research by Ekeuku *et al.* 26, who observed similar reductions in displacement and strain in male rats fed an HCHF diet for 16 weeks, suggesting a decline in bone ductility, or the ability to withstand significant deformation without fracturing. These findings indicate a potential negative correlation between HCHF and bone strength. Kelulut honey supplementation increased bone strength in rats with metabolic syndrome (MetS). This speculation was evidenced by an increase in bone displacement and strain, and a decrease in bone stiffness in the current study. The findings are consistent with previous research by Yudaniayanti *et al.* 41, which showed that three-month-old ovariectomised female rats supplemented with honey (100, 200 and 400 mg/kg) for 12 weeks exhibited increased bone strength.

Bone histomorphometry revealed a lack of significant differences in bone microstructural and cellular indices between study groups. This observation was contradictory to the study of Wong *et al.* 4, which reported significant decreases in Ob.S/BS, accompanied by an increase in ES/BS and a reduction in OS/BS and BV/TV in rats fed with HCHF compared to controls. However, no changes were observed in Oc.S/BS. Since rats in the study of Wong *et al.* 4 were given HCHF diet for a longer time (20 weeks vs 16 weeks in the current study), we speculated that a longer time is needed to achieve significant changes in bone cell numbers and activity, and microstructural deterioration. Besides, since no specific markers staining was used, sensitivity in detecting cellular changes was reduced in this study.

Increased differentiation of bone marrow stem cells into adipocytes could reduce their differentiation into bone-forming osteoblasts 42-44. We observed increased bone marrow adipocytes in HCHF-fed rats compared to controls, supporting findings by Cai *et al.* 45. Kelulut honey reduced bone marrow adipocytes in HCHF-fed rats, suggesting a potential anti-adipogenic effect. Previous studies have established that stingless bees' honey could prevent visceral adipocyte hypertrophy in rats fed with HCHF 46, 47. These findings warrant further investigation into honey's role in mitigating the detrimental effects of HCHF on bone health.

Prolonged hypercaloric intake can negatively impact bone remodelling, leading to bone loss and deterioration of bone structure. Research shows that long-term consumption of a high-fat diet increases reactive oxygen species (ROS), causing oxidative stress and weakening the body's antioxidant defences. This phenomenon disrupts the balance between bone formation and resorption, thereby promoting the production of proinflammatory cytokines, which can interfere with the osteoprotegerin/receptor activator of NFκB ligand balance, creating a pro-osteoclastogenesis environment favouring bone loss 48.

Oxidative stress and a shift from brown adipocytes are suggested to contribute to the expansion of bone marrow adipose tissue, a feature commonly associated with bone loss conditions like osteoporosis 49. Antioxidants may influence bone marrow adiposity by suppressing the formation of adipocytes and promoting their death in human bone marrow 50, 51. Kelulut honey has been shown to possess antioxidant properties, as evidenced by its high levels of phenolic compounds and its ability to reduce oxidative stress 52-54. Kelulut honey did not alter the activity of key antioxidant enzymes (GSH, SOD, CAT) in our study, but it decreased MDA levels significantly compared to control rats. This observation suggests that Kelulut honey reduces oxidative stress by providing exogenous antioxidants rather than elevating the levels or activities of endogenous antioxidant defence. The reduction in oxidative stress might explain the anti-adipogenic and anti-osteoporosis effects of Kelulut honey.

Gene expression analysis revealed a lack of significant difference in osteogenic gene expression between study groups. Our findings were similar to the study by Lange *et al.* 55, whereby male diabetes-prone rats fed with HFD showed no significant difference in expression of *Rankl* and *Ocn* compared to the control rats. Another study by Kushwaha *et al.* 56 reported no significant difference in the expressions of *Runx2*, *Ocn*, *Opg* and *Ctsk* in bone marrow stromal cells derived from male offspring of HFD-fed dams compared to offspring of chow-fed dams. Our findings suggest that bone metabolism may be relatively resilient to short-term metabolic changes. Longer exposure to HFD or more severe metabolic disturbances might be necessary to observe significant changes in osteogenic genes. Our study showed a downregulation in *Pparg* expression after 16 weeks of HCHF intake. This finding was contrary to that of Chen *et al.* 57, whereby *Pparg* expression was upregulated in growing male rats fed with HFD for 4 weeks. This unexpected observation may suggest that extended exposure to excessive lipids could result in lipotoxicity, potentially disrupting PPARG signalling. On the other hand, Kelulut honey did not alter the expression of genes of interest in the study.

Although this study offers valuable information, it has certain shortcomings. Structural histomorphometry, while informative, may not provide the same level of detail as micro-computed tomography. The dosage and duration of Kelulut honey treatment might not have been optimal for inducing significant skeletal changes. Future investigations should explore higher honey doses or extended treatment periods. Moreover, the molecular mechanisms by which Kelulut honey influences skeletal redox status and bone marrow adiposity were not examined. Considering the inflammatory environment created by the HCHF diet, which can adversely affect skeletal health 51, future studies should incorporate markers of inflammation to understand the pathogenesis of HCHF-induced bone loss and the protective effects of Kelulut honey. The metabolic parameters of rats in this study were not covered because they had been covered in a previous publication 46.

## Conclusions

Our findings suggested that rats fed with an HCHF diet exhibited decreased bone flexibility and potentially compromised fracture resistance. However, bone structural and cellular changes were lacking in the HCHF rats. Kelulut honey increased bone displacement and strain and reduced stiffness in HCHF-fed rats, suggesting improved bone flexibility. The HCHF diet also increased adipocytes in the bone marrow, but it was suppressed by Kelulut honey. Kelulut honey supplementation countered this effect, potentially by reducing oxidative stress in the bone marrow microenvironment. Future studies could explore the specific mechanisms by which honey exerts its protective effects and explore the optimal dosage for bone health benefits.

## Figures and Tables

**Table 1:** Primer sequence used in the study.

Gene	Forward Primer	Reverse Primer
*Runx2*	GTTATGAAAAACCAAGTAGCCAGGT	GTAATCTGACTCTGTCCTTGTGGAT
*Rankl*	ACCAGCATCAAAATCCCAAG	TTTGAAAGCCCCAAAGTACG
*Opg*	GTTCTTGCACAGCTTCACCA	AAACAGCCCAGTGACCATTC
*Ctsk*	GCAGCAGAATGGAGGCATTG	TTCAGGGCTTTCTCGTTCCC
*Ocn*	GGTGCAAAGCCCAGCGACTCT	GGAAGCCAATGTGGTCCGCTA
*Pparg*	GTCTCACAATGCCATCAGGT	AGCTGGTCGATATCACTGGA

**Table 2:** List of compounds detected in the honey sample using LC-MS/MS.

No	Retention time (min)	m/z	Adduct	Molecular weight (Da)	PubChem Name	CID	Formula
1	0.485	104.07100	[M+H]+	103.06316	Gamma-aminobutyric acid	119	C4H9NO2
2	0.522	104.10725	[M]+	104.10725	Choline	305	C5H14NO
3	0.472 (0.476)	106.05018 (104.03429)	[M+H]+ ([M-H]-)	105.04234 (105.04213)	Serine	5951	C3H7NO3
4	2.976	118.06545	[M+H]+	117.05761	Indole	798	C8H7N
5	0.69	118.08644	[M+H]+	117.07860	Valine	1182	C5H11NO2
6	0.553	118.08648	[M+H]+	117.07864	Betaine	247	C5H11NO2
7	0.697	136.06166	[M+NH4]+	118.02320	Methylmalonic acid	487	C4H6O4
8	0.499 (0.496)	120.06594 (118.04964)	[M+H]+ ([M-H]-)	119.05810 (119.05748)	Threonine	205	C4H9NO3
9	3.064	122.08775	[M+H]+	121.07991	Phenethylamine	1001	C8H11N
10	1.069	123.04432	[M+H]+	122.03648	Benzoic acid	243	C7H6O2
11	0.651	125.02330	[M-H]-	126.03114	1,2,4-Benzenetriol	10787	C6H6O3
12	21.815	127.03920	[M+H]+	126.03136	4-Hydroxy-6-methyl-2-pyrone	54675757	C6H6O3
13	0.499	130.05013	[M+H]+	129.04229	Pyroglutamic acid	7405	C5H7NO3
14	0.735	130.08636	[M+H]+	129.07852	Pipecolic acid	849	C6H11NO2
15	1.301	132.10193	[M+H]+	131.09409	Isoleucine	6306	C6H13NO2
16	0.489	132.02922	[M-H]-	133.03706	Aspartic acid	5960	C4H7NO4
17	0.581	138.05490	[M+H]+	137.04706	Trigonelline	5570	C7H7NO2
18	2.976	120.08118	[M-H2O+H]+	137.08862	Phenylethanolamine	1000	C8H11NO
19	21.94	143.03418	[M+H]+	142.02634	Kojic acid	3840	C6H6O4
20	3.595	146.06030	[M+H]+	145.05246	Indole-3-Carboxaldehyde	10256	C9H7NO
21	0.499 (0.489)	147.07651 (145.06079)	[M+H]+ ([M-H]-)	146.06867 (146.06863)	Glutamine	5961	C5H10N2O3
22	0.425	147.11292	[M+H]+	146.10508	Lysine	5962	C6H14N2O2
23	0.913	146.11766	[M]+	146.11766	Acetylcholine	187	C7H16NO2
24	0.693	146.04465	[M-H]-	147.05249	N-Acetyl-DL-serine+G31	352294	C5H9NO4
